# Child dietary diversity and food (in)security as a potential correlate of child anthropometric indices in the context of urban food system in the cases of north-central Ethiopia

**DOI:** 10.1186/s41043-020-00219-6

**Published:** 2020-12-02

**Authors:** Amare Molla Dinku, Tefera Chane Mekonnen, Getachew Shumye Adilu

**Affiliations:** 1 Researcher at the Department of Rural Development and Agricultural Extension, College of Agriculture, Wollo University, Dessie, Ethiopia; 2 Researcher at the School of Public Health, College of Medicine and Health Sciences, Wollo University, Dessie, Ethiopia; 3 Researchr at the Department of Plant Science, College of Agriculture, Wollo University, Dessie, Ethiopia

**Keywords:** Food insecurity, Dietary diversity, Urban food system, Child anthropometry, North-central Ethiopia

## Abstract

**Objective:**

To investigate the relation of child dietary diversity and household food insecurity along with other socio-demographic with child anthropometric indices in north-central Ethiopia, an area with a high level of food insecurity and inadequate diet quality.

**Design:**

A community-based cross-sectional study was used.

**Settings:**

The study was conducted in Dessie and Combolcha towns of north-central Ethiopia from April to May 2018.

**Participants:**

Randomly selected 512 mother-child pairs with child’s age range of 6–59 months.

**Results:**

The mean (± SD) scores of weight-for-height/length, height/length-for-age, weight-for-age, and BMI-for-age *Z*-scores were 1.35 (± 2.03), − 1.89 (± 1.79), 0.05 (± 1.54), and 1.39 (± 2.06), respectively. From all anthropometric indicators, stunting and overweight/obesity remained the severe public issues hitting 43% and 42% of the children, respectively. In the model, mothers’ age and education and child’s age, sex, and dietary diversity were significantly related with child height-for-age *Z*-score while place of residence, sex of household head, child’s age, and dietary diversity score were the predictors of child BMI-for-age *Z*-score in the urban contexts of the study area. Nevertheless, food insecurity was not related to any of the child anthropometric indices.

**Conclusion:**

The double burden of malnutrition epidemics (stunting and obesity) coexisted as severe public health concerns in urban settings. Anthropometric statuses of children were affected by multidimensional factors and seek strong integration and immediate intervention of multiple sectors.

## Introduction

Currently, Africa is experiencing rapid demographic changes which are partly driven by the increasing migration of individuals to urban areas and rapid socio-economic transitions [1–3]. Nearly, one third of the population of Africa live in cities, and this figure is expected to be more than one half by the end of 2033 [4]. In East African countries, the average rate of urban population growth projection from 1995 to 2025 was 4.7%, and after 2025, 41.2% of the regional population will be expected to live in urban areas [5, 6]. Similarly, in Ethiopia, nearly 20 million people are currently living in urban areas where the annual urban population growth rate is 4.8%, which is equivalent to the East African average [7]. Such urbanization changes the food habits and lifestyles of the urban population, which placed an additional burden on the nutritional problems of children [8–10]. Due to the changes in people’s food environments and dietary habits, nutrition transition has been occurring in the last decade [9]. This in turn results in a modification in food patterns which are associated with the increasing levels of obesity and nutrition-related non-communicable diseases coexisting with malnutrition [1, 2, 11].

In the early years, diet-related poor child health outcomes in urban areas are increasing and have negative ripple effects on the timing of entry into school educational attainment which ultimately resulting in intergenerational poverty and malnutrition [4]. Thus, this malnutrition prevents children from reaching their full physical and mental potential [12].

Even though the prevalence of stunting and acute malnutrition (wasting or low weight-for-height) in Ethiopia has decreased over the past decade, it remains high, with 38% of children under 5 years stunted and 10% wasted [13]. Stunting is even higher in Amhara regional state with 46% and more persistent in north-central Ethiopia [13, 14]. Recognizing this, the Government of Ethiopia had launched a nutrition program called “Seqota Declaration” to be executed from 2016 to 2030 focused on ending child undernutrition by 2030 [14]. Therefore, the aim of this study was to explore the nutritional status of 6–59-month children who live in urban areas in north-central Ethiopia and to investigate their potential covariates.

## Methods and materials

### Study design, settings, and participants

We employed a cross-sectional study in Dessie and Combolcha towns from April to May 2018. Dessie and Combolcha cities are found in South Wollo Zone, north-central Ethiopia, with elevations between 1842 and 2550 m above sea level, as Combolcha takes the lower elevation. There is more than 58% of the total rainfall in the summer season, while 18% falls in spring and less than 5% of the total occurring during winter. The uneven distribution of rainfall gives rise to a serious shortage of water during the dry season in the area [15]. Most of the time, small business/self-employment and government salary/wages were still the main livelihood activities for most urban households. Considering variations by town with regard to food security conditions, Dessie had the second-highest poor consumption percentage of households (47%) [16].

All children aged between 6 and 59 months that have resided in the study area for the last 6 months were included in the study sample, while any child with a severe medical problem, lack of household head or caregivers, or physical deformity was excluded from the study. The largest sample (512 mother-child pairs) was taken from a study conducted in Ethiopia by considering maternal education as a predictor for child stunting and overweight [17] with the assumptions that 95% of confidence level, 80% of power, 1.7 the odds of being stunting when the mother is not educated, and 24.3% of child stunting among uneducated mothers. Three sub-cities from Dessie and two kebeles from Combolcha town were selected randomly. We conducted a preliminary census in the selected catchments to identify target participants. The samples allocated to the total populations with the eligible study subjects proportionally, and the younger child was selected if more than one child were found in the household.

### Data collection and measurements

A predesigned and pre-tested questionnaire was used to interview the study participants to elicit information on family and child socio-demographic characteristics like residence, religion, type of family, education, occupation of parents, socio-economic condition (household expenditure and wealth index), household food insecurity, child feeding characteristics, and anthropometric measurements.

The questionnaire was standardized to assure the quality and validity of the data and translated into the local language (Amharic) and was re-translated to English. All assessment team members were able to administer the questionnaires properly; a total of 5 days of rigorous training of enumerators and supervisors was given by the three authors. Before the actual data collection work, data collectors and supervisors carried out role-play practices and they filled the pre-test activities in the community other than target areas. Data collectors were responsible for filling out the data using mobile devices while supervisors checked the completeness and correctness of the filled data before sending it to the researchers. At the end of every data collection day, each questionnaire was examined for completeness and consistency by the supervisors and finally cross-checked by the researchers. A regular adjustment has been made for anthropometric measurements in each circumstance.

The household socio-economic status (SES) was parameterized by the principal component analysis (PCA) method using house properties confirmed by the questionnaire: property owned, source of drinking water, type of toilet facility, and type of flooring, wall material, and roof material. The score in the first PCA component was used as an asset index of SES status for each household [18], and households were categorized into tertiles as poor, medium, and rich. The household food security status was assessed using the Household Food Insecurity Access Scale (HFIAS), and households were classified as food secure if it had not experienced any food insecurity conditions or had rarely worried about not having enough food, whereas food-insecure households were categorized as mild, moderate, and severe in accordance with the guidelines [19]. For data validation, Cronbach’s alpha coefficient, which is a measure of the internal consistency of a scale, was used to confirm the reliability of the HFIAS and the household SES measure. An alpha value of more than 0.7 indicated that the measure was acceptable.

Child dietary assessment was done based on the procedure recommended by the Food and Agriculture Organization (FAO) [20]. Mothers or caregivers were asked whether the child consumed more than a spoonful of the seven food groups (namely, cereals, tubers and roots, legumes and nuts, vitamin A-rich fruits and vegetables, flesh foods, milk and milk products, eggs, and other fruits and vegetables) within the past 24 h recall. The child food groups were developed based on the food items recommended in the Infant and Young Child Feeding (IYCF) guidelines. The total dietary diversity score was generated with the response of “yes” and “no” for each child. In accordance with the IYCF guidelines, a child’s DDS was categorized as poor and good [21].

#### Child anthropometry

Child weight and length/height were taken by following critical and meticulous procedures. Ages were also recorded from immunization cards, direct probing of mothers, or birth certificates. The weight of children was taken using an electronic digital weight scale and recorded in kilograms to the nearest 0.1 kg [22] and with light clothes and no shoes. Two measurements were recorded for each child, and the average result was taken. In every instance of measurement, the scale was checked for its reading and calibration. It was also standardized with 2 kg iron rod before taking the measure. The length/height of the child was also documented twice. The length was measured for children less than 24 months (child unable to stand erectly or < 85 cm) in recumbent position using wood-made sliding length board with the help of two examiners. For children greater than 24 months, height was measured using a sliding height board in Frank fret position and recorded in centimeters to the nearest 0.1 cm [22]. During this procedure, hats and shoes were removed, and the gentle pressing of hair has been made. The data were collected using a mobile data collection tool called Open Data Kit (ODK), and the collected data was directly sent to the KoBo Toolbox account created by the researchers. The daily data collected and submitted by the data enumerators were checked and cleaned by the researchers. Finally, the collected data were exported to STATA version 15 and made ready for data analysis. Standardization of measurements has been carried out, and the coefficient of variation was kept minimal (< 3%) for weight and height measurements.

### Data management and analysis

The data were cleaned and prepared for analysis, and STATA version 15 (StataCrop LLC, College Station, TX 77845, USA) was used to present the summary results and inferential statistics. Exploratory data analyses were done to identify missing values, influential outliers, and normality of data for both outcome and explanatory variables. Anthropometric data were exported to WHO Anthro Software version 3.2.2 to generate anthropometric indices for weight-for-length/height *Z*-score (WHZ), height/length-for-age *Z*-score (HAZ), weight-for-age *Z*-score (WAZ), and BMI-for-age *Z*-score (BAZ). Child nutritional status was determined using the above indices where each of the indices < − 2 SD is categorized as wasted, stunted, underweight, and thin. The child overnutrition was also defined when BAZ score between + 2 and + 3 SD and greater than + 3 SD reflecting the presence of overweight and obesity, respectively. We omitted outliers for WHZ and BAZ when less than − 5 and greater than + 5 and for HAZ and WAZ when the score less than − 6 and greater than + 6, respectively [22]. We fitted a generalized linear model (GLM) to declare the presence of significant associations between anthropometric indices (WHZ, HAZ, WAZ, and BAZ) and different explanatory variables. Maximum likelihood estimation was used to estimate the parameters. We checked the assumption for GLM for independently distributed outcome variables; not more than 20% of the expected cells had less than 5 for goodness-of-fit measures and the presence of the relationship between the transformed response in terms of the link function and the explanatory variables. To assess confounding, factors were included in the model based on biological plausibility and known epidemiological predisposing factors such as socio-demographic characteristics, socio-economic status, food insecurity, and child feeding practices.

### Ethical consideration

During data collection, a letter of ethical clearance was collected from the Wollo University, College of Medicine and Health Sciences. Particularly, the institutional health research ethics review committee was consulted about the importance of the research to the community and the harms that would occur during data collection. An official letter has been written for each city administrator and health office where the data were taken. In addition, informed verbal consent was obtained from each client, and confidentiality was maintained by giving codes for each respondent rather than recording their names. Data collectors were informed that clients have full right to discontinue or refuse to participate in the study.

## Results

### Socio-demographic features of participants

A total of 506 mothers with child pairs were provided a complete response that gives a response rate of 98.8%. The mean age of the household head was found to be 32.2 (± 8.5 SD) years, and 355 (70.2%) of the study subjects were male household headed. Regarding the marital status of the participants, majority (459 (90.7%)) of them were married (Table 2). The average family sizes per household were 4.7 (± 1.9 SD) (Table 1).

**Table 1:** Distribution of socio-demographic characteristics of study participants in Dessie and Combolcha towns, north-central Ethiopia May 2018

Variables		Frequency (%)
Sex of household head	Male	355 (70.2)
	Female	151 (29.8)
Age of household head (in years)	< 25	63 (12.5)
	25–30	205 (40.5)
	31–35	108 (21.3)
	36–40	63 (12.5)
	> 40	67 (13.2)
City	Dessie	358 (70.8)
	Combolcha	148 (29.2)
Village	Aba Kolba	9 (1.8)
	Erfo	139 (27.5)
	Arada	77 (15.2)
	Hotie	248 (49)
	Buanbua Wuha	33 (6.5)
Maternal educational status	Formal	440 (89)
	Informal	28 (5.5)
	Non-educated	38 (7.5)
Total average expenditure (in ETB)		4916 ± 2766.9
Estimated average household monthly income (in ETB)		5644.9 ± 3933.3
Socio-economic status	Poor	173 (34.3)
	Medium	190 (37.7)
	Rich	141 (28.0)
Source of income	Employment only	170 (33.6)
	Casual labor only	79 (15.6)
	Non-agricultural only	83 (16.4)
	Multiple sources	174 (34.4)
Access for latrine	Yes	288 (56.9)
	No	218 (43.1)

### Household food security and socio-economic characteristics of participants

About the sources of drinking water, majority (445 (87.9%)) of the respondents had a private pipeline in their compounds while the rest used from communal pipe and spring water sources. On the other side, about two fifth (43.1%) of the study participants used a latrine outside their home that might be from communal latrine or open defecation (Table 1).

**Fig. 1:**
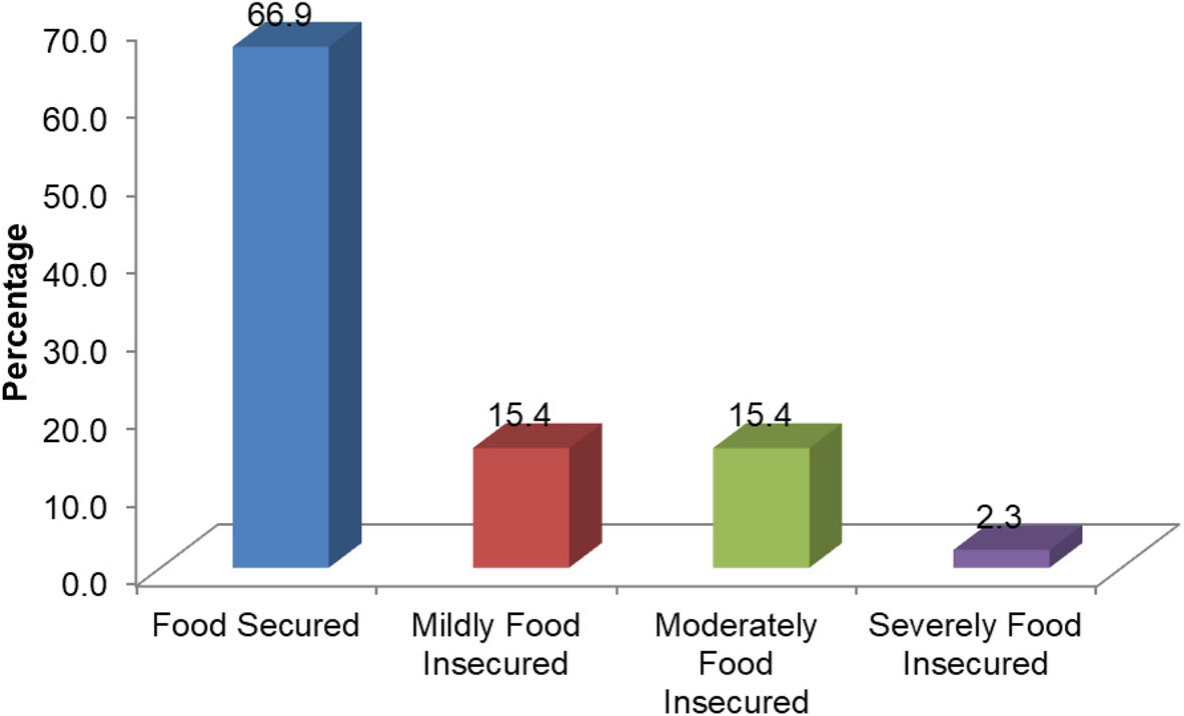
Food security condition among households living in Dessie and Combolcha towns, north-central Ethiopia May 2018

The study reported that from the total of study subjects, 168 (33.1%) of them were found in households that experienced any form of food insecurity. The extent of food insecurity was much worse among households who are female-headed than male-headed (57.3% vs 22.9%, *p* < 0.0001) (Fig. 1), and it was more than twofolds higher in households of non-educated mothers (76.3%) compared to educated mothers (28.5%) (*p* < 0.0001).

### Child feeding practices

**Fig. 2:**
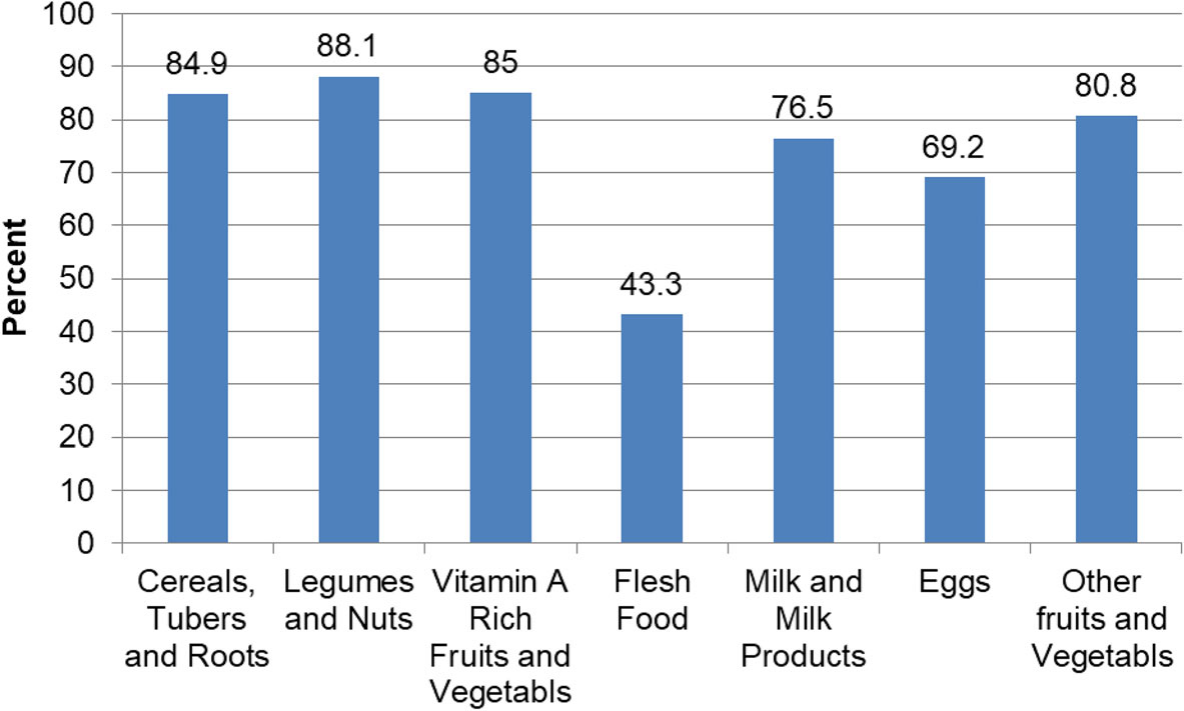
Distribution of food groups consumed by children during 24 h prior to the data collection period in north-central Ethiopia

Among all children, 449 (88.7%) of them had good dietary diversity who consumed four and above food groups. Of the 24 h recall, the most common or staple food group consumed by children was pulses and nuts followed by cereals. However, consumption of iron-, zinc-, and vitamin A-rich animal source foods was eaten by less than 50% of the children (Fig. 2).

When compared to child’s diversified eating habits across the household socio-economic classes, good dietary diversity was higher among children from poor classes compared to rich socio-economic category (62% vs 22%, *p* < 0.0001), while it was higher among children from food-secure households compared to an insecure one (69.4% vs 24%, *p* < 0.0001). Additionally, children from educated mothers, married women, and male-headed households had at least twofolds of better dietary diversity as compared to those from non-educated mothers, unmarried women, and female-headed households, respectively (Table 2).

**Table 2:** Associations of child dietary diversity scores and food insecurity condition with socio-demographic features of the respondents in north-central Ethiopia May 2018

Variables		Child dietary diversity score (CDDS)				Food security status		
		Poor (0–3)	Medium (4–5)	Good (6–7)	***p*** value	Food secure	Food insecure	***p*** value
Sex of household head	Male	23 (6.48)	112 (31.54)	220 (61.97)	0.0001	273 (77.12)	81 (22.9)	0.0001
	Female	33 (22)	62 (41.33)	55 (36.67)		64 (42.67)	86 (57.33)	
Food security situation	Food secure	10 (3%)	93 (27.6%)	234 (69.4%)	0.0001			
	Food insecure	46 (27.5%)	81 (48.5%)	40 (24%)				
Sex of child	Male	20 (8.9%)	69 (30.8%)	136 (61.3%)	0.253	164 (74.2%)	58 (25.8%)	0.002
	Female	36 (12.9%)	105 (37.6%)	139 (50.5%)		170 (60.9%)	109 (39.1%)	
Mothers’ education	Formal	37 (7.5%)	150 (34.2%)	256 (58.3%)	0.0001	255 (78%)	72 (22%)	0.0001
	Informal	4 (14.3%)	15 (53.6%)	9 (32.1%)		9 (60%)	6 (40%)	
	Non-educated	19 (50%)	9 (23.7%)	10 (26.3%)		9 (75%)	3 (25%)	
Marital status	Divorced	9 (31%)	10 (34.5%)	10 (34.5%)	0.0001	2 (20%)	8 (80%)	0.0001
	Married	40 (8.7%)	158 (34.4%)	261 (56.9%)		320 (69.9%)	138 (30.1%)	
	Unmarried	2 (28.6%)	3 (42.9%)	2 (28.6%)		5 (71.4%)	2 (28.6%)	
	Widowed	5 (50%)	3 (30%)	2 (20%)		10 (34.5%)	19 (65.5%)	
Wealth index	Poor	9 (5.2%)	57 (33.1%)	106 (61.6%)	0.0001	73 (36.3%)	128 (63.6%)	0.0001
	Medium	3 (1.6%)	51 (26.8%)	136 (71.6%)		85 (79.4%)	22 (20.6%)	
	Rich	44 (31.2%)	66 (46.8%)	31 (22%)		177 (91.2%)	17 (8.8%)	

### Child nutritional status

From a total of 316 children who had complete data for weight-for-height *Z*-score, 17 (5.4%) of them were wasted and boys seemed to be more affected than girls (5.8% vs 4.3%). Similar results were found between WHZ and BAZ to illustrate the burden of acute malnutrition among children. By BAZ scores (< − 2 SD), 5.2% (95% CI 3.2, 8.4) of children were acutely malnourished. Regarding the degree of chronic undernutrition from 340 children, about two of every five children were stunted (42.9%; 95% CI 37.4, 48.6), and of which 21.4% (95% CI 17.4, 26.2) and 25.2% (95% CI 20.9, 30.2) were moderately and severely stunted, respectively. On the other hand, the proportion of children who had either wasting or stunting or both was 10.5% (95% CI 7.5, 14.5). The co-existence of a double burden of malnutrition in the area was observed. Of all children, 42.2% (95% CI 36.8, 47.9) of them were overweight and/or obese. With the BAZ score of greater than 3, and between 2 and 3 of SD, 27.9% (95% CI 23.2, 33.1) and 15.2% (95% CI 11.7, 19.7) of children were categorized as having obesity and overweight, respectively.

The mean of WHZ, HAZ, BAZ, and WAZ scores (SD) was 1.35 (± 2.03), − 1.89 (± 1.79), 1.39 (± 2.06), and 0.05 (± 1.54), respectively. The presence of a linear relationship between different anthropometric indices and many covariates was examined (Table 3). Child age (*r* = 0.18, *p* = 0.02), child dietary diversity (*r* = 0.3, *p* < 0.0001), and animal source food score (*r* = 0.23, *p* < 0.0001) were positively correlated with child WHZ score, but the food insecurity score (*r* = − 0.11, *p* < 0.04) was negatively correlated, while HAZ score was positively correlated with age of household head (*r* = 0.17, *p* < 0.001) but negatively correlated with child dietary diversity scores (*r* = − 0.18, *p* < 0.001), child age (*r* = − 0.2, *p* < 0.0001), and animal source food (*r* = − 0.15, *p* < 0.006).

**Table 3:** Correlation of child anthropometric indices with selected socio-economic and demographic variables and child feeding practices in north-central Ethiopia May 2018

Variables		WHZ	HAZ	BAZ	WAZ	MUACZ
**Age of HHH (years)**	Correlation coefficient	− 0.06	0.17	− 0.065	0.144	0.095
	*p* value	0.29	**0.001**	0.25	0.002	0.04
**Family size**	Correlation coefficient	0.03	0.03	0.02	0.10	0.06
	*p* value	0.59	0.60	0.73	0.02	0.20
**Age of child (in months)**	Correlation coefficient	0.18	− 0.20	0.14	− 0.19	0.10
	*p* value	**0.002**	**0.0001**	0.011	0.0001	0.025
**Wealth index score**	Correlation coefficient	− 0.07	0.03	− 0.07	− 0.09	− 0.08
	*p* value	0.19	0.53	0.20	0.04	0.09
**Food insecurity score**	Correlation coefficient	− 0.11	0.11	− 0.13	− 0.10	− 0.05
	*p* value	**0.04**	0.050	0.025	0.03	0.25
**Child dietary diversity score**	Correlation coefficient	0.30	− 0.18	0.31	0.20	0.11
	*p* value	**0.0001**	**0.001**	0.0001	0.0001	0.014
**Animal source food score**	Correlation coefficient	0.23	− 0.15	0.24	0.16	0.17
	*p* value	**0.0001**	**0.006**	0.0001	0.0001	0.0001
	*N*	317	340	315	474	480

*HHH* household head

Source: own survey, 2018

### Determinants of child nutritional status

Potential candidate covariates were included in the generalized linear model to identify their level of statistical significance with different child anthropometric indices. Anthropometric indices WHZ, HAZ, WAZ, and BAZ scores as dependent variables were tested with the area of child residence, sex of household head, family size, age of mother, education of mother, marital status, age and sex of child, wealth index score, food insecurity, child dietary diversity score, and animal source food score. After adjustment of the model, child WHZ had significant positive or negative associations with the place of residence [*β* = − 1.33; 95% CI − 1.78, − 0.89], sex of household head [*β* = − 0.97; 95% CI − 1.56, − 0.35], child age [*β* = 0.31; 95% CI 0.13, 0.50], and child DDS [*β* = 0.48; 95% CI 0.28, 0.69]. When a unit increase in child dietary diversity score, the child WHZ score increased by about 0.5. Similarly, WAZ has shown significant positive associations with child dietary diversity score and age of mother at [*β* = 0.26, 95% CI 0.12, 0.39] and [*β* = 0.01; 95% CI 0.001, 0.03] successively and negative significant associations with child place residence [*β* = − 0.24; 95% CI − 0.34, − 0.13] and child age [*β* = − 1.03; 95% CI − 1.33, − 0.73].

In addition, HAZ score showed a significant positive association with age of mother [*β* = 0.03; 95% CI 0.01; 0.05], education of mother [*β* = 0.66; 95% CI 0.03, 1.29], sex of child [*β* = 0.50; 95% CI 0.13, 0.87], and animal source food score [*β* = 0.30; 95% CI 0.01, 0.58]. But it was negatively correlated with wealth index, child age, and child dietary diversity score at *β* = − 0.02, 95% CI − 0.07, 0.03; *β* = − 0.25, 95% CI − 0.45, − 0.06; and *β* = − 0.36, 95% CI − 0.53, − 0.20, respectively (Table 4). For instance, the study indicated that as the child dietary diversity score increased by one unit, child linear growth decreased by about 0.5 and showed moderate strength of association.

**Table 4:** Generalized linear model identifying factors influencing child nutrition status in north-central Ethiopia May 2018

Explanatory variables	Child malnutrition			
	WHZ, *β* (95% CI)	HAZ, *β* (95% CI)	WAZ, *β* (95% CI)	BAZ, *β* (95% CI)
**City** (0 = Combolcha, 1 = Dessie)	− 1.33 (− 1.78, − 0.89)***	0.21 (− 0.19, 0.61)	− 1.03 (− 1.33, − 0.73)***	− 1.23 (− 1.7, − 0.78)***
**Sex of household head** (0 = female, 1 = male)	− 0.97 (− 1.56, − 0.35)**	0.30 (− 0.25, 0.87)	0.18 (− 0.50, 0.14)	− 0.07 (− 1.36, − 0.08)*
**Family size**	− 0.004 (− 0.17, 0.16)	0.02 (− 0.06, 0.12)	0.04 (− 0.03, 0.11)	− 0.02 (− 0.19, 0.15)
**Age of mother**	− 0.01 (− 0.04, 0.02)	0.03 (0.009, 0.05)**	0.01 (0.001, 0.03)*	− 0.01 (− 0.05, 0.01)
**Education of mother** (1 = formal, otherwise = 0)	− 0.19 (− 0.89, 0.51)	0.66 (0.03, 1.29)*	0.18 (− 0.24, 0.60)	− 0.54 (− 1.25, 0.17)
**Marital status** (1 = married, otherwise = 0)	0.23 (− 0.5, 1.03)	0.08 (− 0.65, 0.82)	0.21 (− 0.29, 0.70)	0.33 (− 0.49, 1.14)
**Wealth index**	0.02 (− 0.04, 0.07)	− 0.02 (− 0.07, 0.03)	− 0.004 (− 0.03,0.03)	0.02 (− 0.03, 0.08)
**Food insecurity** (0 = secure, 1 = insecure)	0.08 (− 0.43, 0.61)	− 0.09 (− 0.56,0.37)	0.02 (− 0.31, 0.35)	0.21 (− 0.32, 0.75)
**Child age**	0.31 (0.13, 0.50)**	− 0.36 (− 0.53, − 0.20)***	− 0.24 (− 0.34, − 0.13)***	0.23 (0.04, 0.42)*
**Sex of child** (1 = male, 0 = female)	− 0.13 (− 0.55, 0.28)	0.50 (0.13, 0.87)**	0.15 (− 0.11, 0.41)	− 0.25 (− 0.68,0.17)
**Child dietary diversity score**	0.48 (0.28, 0.69)***	− 0.25 (− 0.45, − 0.06)**	0.26 (0.12, 0.39)***	0.48 (0.26, 0.69)***
**Animal source food**	− 0.21 (− 0.52, 0.09)	0.30 (0.01, 0.58)*	0.04 (− 0.15, 0.24)	− 0.22 (− 0.54, 0.09)

Significance at *p* value of *< 0.05, **0.01, and ****0.001

Child anthropometric index by BAZ score was negatively affected by child place of residence [*β* = − 1.23; 95% CI − 1.7, − 0.78] and sex of household head [*β* = − 0.07; 95% CI − 1.36, − 0.08], but it was also positively affected by child’s age [*β* = 0.23; 95% CI 0.04, 0.42] and child DDS [*β* = 0.48; 95% CI 0.26, 0.69]. In terms of residence, a child who lived in Dessie showed a decreased BAZ score of 1.2 points as compared to a child who lived in Combolcha. However, as a child’s dietary diversity score increased by one unit, the child’s BAZ score also increased by 0.5 scores (Table 4).

## Discussion

The study revealed the double burden of malnutrition in the context of the urban food system remained a major public health challenge. The degree of chronic undernutrition and overnutrition posed severe major public concern, in which about 43% and 42% of children in Dessie and Combolcha towns, respectively, were stunted and overweight and/or obese. Given that the mean (± SD) HAZ and BAZ scores were − 1.89 (± 1.79) and 1.39 (± 2.06), respectively, a significant number of children were categorized as stunted and overweight/obese. In the urban settings, the rate of malnutrition among under-five children was persistently higher, and the nutrition transition phenomenon impacted the health of children in which the rate of obesity has been radically progressed and that ultimately attributed to many life-threatening non-communicable diseases including diet-related cardio-metabolic risk and cancer [6, 23, 24]. The prevalence of chronic undernutrition in these urban settings was much higher than from studies conducted in urban settings of Ethiopia in Arba Minch (18.7%) [25], Addis Ababa (19.6%) [17], Kersa district (8.9%) [26], rural Kenya (32%) [27], South Africa (35%) [28], and urban cities of South Africa, Umlazi (28%), Rietvlei (20%), and Paarl (17%) [4]. However, it was less than the findings in rural and pre-urban areas in Ethiopia such as Humbo districts (75%) [29], Merhabete district (52.4%) [30], Libo-kemekem district (49.4%) [31], and Lalibela town (47.3%) [32]. On the other side, the finding was in line with the national prevalence of stunting from EDHS 2016 (38.39%) [33], (37%) [34] reports, Mecha (37.9%) [35] and Arba Minch Health and Demographic Surveillance Site (41.9%) [36], urban poor settings of Kenya (46%) [27], and Dar-es-Salaam, Tanzania (43%) [37]. In addition to the high burden of stunting in the study settings, the rate of overweight/obesity among under-five children became a crosscutting issue, in which about two of every five children were affected by overweight/obesity as illustrated by the current study. The finding was much higher than reports from Bahir Dar (6.9%) [38]; Dire Dawa (14.7%) [39]; Beijing, China (18.7%) [40]; the urban poor setting of Nairobi, Kenya (9%) [27]; and urban, rural, and mountain areas of Italy (21.06%) [41]. The discrepancies might be due to the prolonged time difference between the current and the previous studies, the rapid growth of urbanization, population density and impact of nutrition transition, a shift to higher energy, ultra-processed food sugar, and saturated fat intake.

In the generalized linear model, after adjustments, child’s various anthropometric indices in the study area had been influenced by socio-demographic features, socio-economic status, and child feeding practices. But it was not affected by the urban food insecurity status of the household. Child linear growth (literally called stature or HAZ score) were significantly associated with mothers’ or caregivers’ age and education, sex and age of the child, child DDS, and ASF, while the child’s mean BAZ score was affected by the sex of household head, age of the child, and child DDS. Furthermore, children with a higher score of animal source food were less likely to have shorter statures.

Child diet diversity was inversely and significantly associated with child HAZ score, and it was contradicted with the findings in rural areas of Kenya and East and West Gojjam Zones of Amhara region, Ethiopia, implying that DDS was a proxy measure of childhood stunting [42, 43]. However, in another study conducted in Ethiopia, DDS had no indicated the presence of association with HAZ scores [17, 36]. This could be the fact that the higher the diversity score may not be a guarantee for a higher mean HAZ score unless the food groups consumed may not include essential nutrients and the small sample size may affect the direction of the association in the current study. Unless the consumption in the study setting is not usual, 24 h recall might not reflect the actual relation between dietary diversity and chronic undernutrition. However, evidences showed that usual optimal feeding practices ultimately affect child growth and nutritional status positively [44]. As maternal age increases and mothers became more educated, the mean HAZ score also increased, that is, the child is less likely to become stunted. The finding was also congruent to the reports from EDHS 2016 [33], Northwest Ethiopia [35], Ecuador [45], South Africa [4, 46], five high-income countries (the USA, the UK, Australia, the Netherlands, and Sweden) [47], Bangladesh [48], and China [49]. It is clear that as mothers get older, they experienced many lived skills and learned from their previous caring practices and educated mothers are also change-makers, innovators and capable enough to transfer knowledge and skills gained, and easily understand child feeding principles and the impact of malnutrition on their child’s health [17, 23].

The child mean HAZ score was negatively affected as a child became aged which was compatible with shreds of evidence from China [49], Gojjam of Ethiopia [43], Southern Ethiopia [29], data from multilevel analysis of EDHS 2016 [33], northwest Ethiopia [30, 35], and Lalibela of Ethiopia [32], and being male in gender positively related with HAZ score similar with reports in Ethiopia [43] but in contrast to the studies from South Africa depicted that males are disproportionately affected by stunting [46], Western China [49]. These may be due to the fact that when the child gets older, they rely on the quality of complementary food, become independent and fed themselves, and get less attention given from parents.

The child BAZ score had shown a significant relation to child dietary diversity score and supported by studies conducted in Bahir Dar [38] and South Africa [46], but a study conducted in South Africa reflected the inverse association between DDS and BAZ [28] and Southern Ethiopia [36]. The possible justification for the higher mean of BAZ score among children with higher DDS is that the inclusion of a variety of foods from different groups did not contribute to optimum nutrition unless the balance or proportion of foods was taken into consideration. The energy contribution from energy-yielding nutrients should be within the recommended energy range and consumption of Western diet (processed, trans-fats, saturated fats) which leads to positive energy balance and leads to prompt adipose-tissue accumulation, must be restricted to combat the growing epidemic of obesity.

Children from male-headed households and reside in Dessie [50] were negatively related to child BAZ score, but child age had a positive relationship with BAZ score [50]. Even though there is no clear justification with the above relationships, in areas where maternal empowerment was less emphasized and decision-making power left only for males, maternal autonomies for child’s feedings are affected. So, mothers or caregivers will face difficulty in providing food for their children for what they want and depend on the willingness of husbands. A child from Dessie was thinner than a child from Combolcha, and this may be due to Dessie is the third city by population-density in Ethiopia and recently covered by an urban-food security program following Addis Ababa. Many of the households faced chronic food insecurity, and the quality of dietary diversity was highly compromised in the area. In this study, when a child’s age increases, the probability of being obese is higher. This may be related to frequent exposure of children to sweetened foods, soft drinks, and preference for processed foods which all are highly accessible in the area.

Food insecurity and socio-economic status of households in the urban context of the current study did not show association with any of the child anthropometric indicators which were similar to the reports from rural Cambodia [51], rural community of Southeastern Kenya [52], and urban poor children of Kenya [53]. A scoping review suggested that household food insecurity may not be associated with height inequalities among children in Canada and the USA and provided insufficient evidence to determine whether food insecurity is or is not associated with children’s height in these countries [54]. Nevertheless, some other studies from the Tamale Metropolis of Northern Ghana, poor rural areas of China [55], and León, Nicaragua [56] agreed that children from food-insecure households were found in a higher probability of being stunted or had lower mean HAZ scores. But a study from Ecuador implicated that food insecurity affects child stature, but does not increase calorie intakes that lead to obesity [57].

This study has several limitations. It does not consider potential mediator variables such seasonal variability that may affect food insecurity and feeding practices, environmental factors, host factors, physical activity, and other lifestyle factors that cumulatively impact child stature and fat body composition of children. We cannot also discount the effect of nutrient intakes and details of food frequency for processed foods and fruits and vegetables. We excluded some data due to missing anthropometric measurements that may have introduced a selection bias (if not missing at random), and may thus have affected both the internal validity and the representativeness of the findings in the broader Ethiopian context.

## Conclusion and recommendation

The study concludes that the co-existence of the double burden of malnutrition epidemics in urban settings. The presence of different forms of malnutrition is characteristics, rapid urbanization, population growth, and high inequalities. Both forms of malnutrition are calling for immediate interventions due to their severe public health concerns. Mothers’ age and education, child’s age, sex, and dietary diversity were the potential determinants of child’s linear growth while the area of residence, sex of household head, child’s age, and dietary diversity were the predisposing correlates of a child’s body fat composition. The dual epidemic of undernutrition and overweight/obesity requires differential policy inputs in metropolitan areas. Very little obesity prevention interventions targeting children have been effective and a comprehensive, multifaceted strategy tackling diet, physical inactivity, coupled with psychosocial support and local food environment change may prove more effective. Nutrition policies tackling child obesity must promote household nutrition security and healthy growth, decrease overconsumption of nutrient-poor foods, better shield children from the increasingly pervasive marketing of energy-dense, nutrient-poor foods and sugar-sweetened beverages as well as reduction of growing physical inactivity [47]. The cost of undernutrition in Ethiopia remained high, and it will be very complicated if childhood obesity is also added as a health challenge. Effective public health planning and contextually tailored interventions are required at the subnational level to address this challenge. Multi-sectoral action may work best given the complex nature of the prevailing circumstances in urban poor settings. Further research is needed to understand the pathways to this coexistence and to test the feasibility and effectiveness of context-specific interventions to curb associated health risks.

## Data Availability

The manuscript contains the all the data needed, and we can provide the dataset upon any request.

## Abbreviations

ASF: Animal source food
BAZ: BMI-for-age *Z*-score
CL: Confidence level
DDS: Dietary diversity score
EDHS: Ethiopian Demographic and Health Survey
FAO: Food and Agriculture Organization
GLM: Generalized linear model
HAZ: Height-for-age *Z*-score
HFIAS: Household Food Insecurity Access Scale
IYCF: Infant and Young Child Feeding
MUACZ: Mid-upper arm circumference for age *Z*-score
PCA: Principal component analysis
SD: Standard deviation
SES: Socio-economic status
WAZ: Weight-for-age *Z*-score
WHO: World Health Organization
WHZ: Weight-for-height *Z*-score
